# Epidemiology of Symptomatic Human Metapneumovirus Infection in the CASCADIA Community-Based Cohort — Oregon and Washington, 2022–2024

**DOI:** 10.15585/mmwr.mm7411a2

**Published:** 2025-04-03

**Authors:** Mila Shakya, Helen Y. Chu, Janet A. Englund, Melissa Briggs-Hagen, Marco Carone, Jennifer L. Kuntz, Tina Lockwood, Claire M. Midgley, Mark A. Schmidt, Lea Starita, Ana A. Weil, Ryan E. Wiegand, Allison L. Naleway, Ian D. Plumb

**Affiliations:** ^1^Epidemic Intelligence Service, CDC; ^2^Coronavirus and Other Respiratory Viruses Division, National Center for Immunization and Respiratory Diseases, CDC; ^3^Department of Medicine, Division of Allergy and Infectious Diseases, University of Washington, Seattle, Washington; ^4^Department of Pediatrics, Seattle Children’s Research Institute, Seattle, Washington; ^5^Department of Biostatistics, University of Washington, Seattle, Washington; ^6^Center for Health Research, Kaiser Permanente Northwest, Portland, Oregon; ^7^Brotman Baty Institute for Precision Medicine, University of Washington, Seattle, Washington; ^8^Department of Pathology, University of Washington, Seattle, Washington; ^9^Genome Sciences, University of Washington, Seattle, Washington.

**Summary**

**What is already known about this topic?**

Human metapneumovirus (hMPV) causes substantial respiratory illness worldwide. However, information on the epidemiology of symptomatic infection is limited, particularly outside of health care settings.

**What is added by this report?**

In this community cohort study including participants aged 6 months–49 years, average incidence of symptomatic hMPV infection was 7.5 per 100 persons per year. Incidence was highest during January–March and among children aged 2–4 years, and clustered in households. Although most infections caused mild illness, 27% were associated with absenteeism from work, school, or a child care facility.

**What are the implications for public health practice?**

Better understanding of the epidemiology of hMPV infection in the community can guide clinical testing and future strategies for prevention and treatment.

## Abstract

Human metapneumovirus (hMPV) is an important cause of respiratory illness. However, information about hMPV incidence, patient characteristics, and symptoms outside hospital settings is limited. During June 2022–March 2024, participants aged 6 months–49 years who were enrolled in the CASCADIA community-based cohort study submitted weekly illness surveys and nasal swabs, and completed follow-up illness surveys. Swabs collected 0–3 days before reporting new or worsening symptoms were tested for hMPV and other respiratory viruses by multiplex polymerase chain reaction. Incidence was analyzed using an exponential survival model. Among 3,549 participants, 306 had symptomatic hMPV infection, representing an average of 7.5 cases per 100 persons per year (95% CI = 6.7–8.4). Incidence was highest during January–March (adjusted hazard ratio [aHR] = 4.3; 95% CI = 3.0–6.0) compared with October–December, and among those aged 2–4 years (aHR = 5.8; 95% CI = 3.8–9.0) compared with those aged ≥40 years. The most frequently reported symptoms were cough (80.4%) and nasal congestion (71.9%). Among 252 (82.4%) participants who completed a post-illness follow-up survey, 68 (27.0%) missed work, school, or child care facility attendance. Together, these findings indicate that hMPV is a common cause of respiratory illness during late winter to spring, particularly among young children, and frequently disrupts daily activities. Understanding hMPV epidemiology can guide surveillance definitions, clinical testing, and prioritization of prevention strategies.

## Introduction

Infection with human metapneumovirus (hMPV), a member of the *Pneumoviridae* family, causes respiratory illness among children and adults, leading to substantial burdens of hospitalizations worldwide (*1*). However, health care providers do not routinely test for hMPV in most clinical settings, treatment remains supportive, and information about the epidemiology of symptomatic infections outside health care settings is limited. Although no currently approved vaccines or treatments are available for hMPV in the United States, several such products are under development (*2*). This report summarizes the epidemiology of symptomatic hMPV infection among participants in a cohort study designed to characterize respiratory virus infections in the community.

## Methods

### Data Source

During 2022–2023, Oregon and Washington residents aged 6 months–49 years were invited to enroll in CASCADIA, a prospective, community-based cohort study (*3*). Participants in households with multiple members were prioritized for enrollment, although participation was not required for all household members. During June 2022–March 2024, participants completed an enrollment survey, followed by weekly nasal swabs (collected by participants or caregivers) and weekly electronic surveys that asked whether participants had experienced any new illness. Swabs were routinely tested for SARS-CoV-2, respiratory syncytial virus, and influenza virus; swabs that tested positive for these viruses or that were associated with new illness were also tested for hMPV and other respiratory pathogens using multiplex polymerase chain reaction (PCR)* (*3*).

### Identification of hMPV Infections

A symptomatic hMPV infection was defined as the occurrence of any new or worsening symptoms^†^ reported by a participant 0–3 days after collection of an hMPV-positive nasal swab, provided that the swab was collected 2 days before through 7 days after reported illness onset.^§^ Participants who reported new illness were invited to complete a 14-day follow-up survey to assess health care usage and absenteeism from work, school, or child care facility.

### Calculation of hMPV Incidence and Identification of Factors Associated with Infection

Incidence of symptomatic hMPV infection was calculated as cases per 100 persons per year of follow-up. Follow-up time was included between qualifying survey responses for each participant. To enable ascertainment of the analysis outcome, qualifying responses were defined as those indicating no new illness, or new illness with a nasal swab collected 0–3 days before the survey, provided that the swab was also collected 2 days before through 7 days after illness onset and had multiplex PCR test results. Follow-up time was censored in the event of a gap between qualifying survey responses of >14 days, and from onset of any symptomatic hMPV infection until 30 days later.

To assess factors associated with risk of symptomatic hMPV infection over time, incidence was modeled as a survival function using an exponential distribution. A random effects term (θ) with a gamma distribution (a continuous probability distribution used to model time-to-event data) was used to allow incidence to cluster within households; household clustering was assumed to occur if θ was greater than zero, using a significance threshold of p<0.05.

The model was adjusted for age group, sex, race and ethnicity, year, quarter of the year,^¶^ reported household size, household income, and presence of underlying health conditions,** all defined a priori.^††^ Detections of hMPV alone and codetections with other respiratory pathogens were included in the main analysis.

Among all infections with detection of hMPV, univariable logistic regression was used to compare differences in symptoms, health care usage, and illness-related absenteeism between persons aged <18 and ≥18 years.^§§^ To exclude symptoms or illnesses with codetected pathogens, analyses were repeated and restricted to the subset of participants with detections of only hMPV. Data were analyzed using Stata (version 18.5; StataCorp). This study was reviewed and approved by the Kaiser Permanente Northwest Region Institutional Review Board; CDC deferred to this institution’s determination. All participants in the study provided written consent.^¶¶^

## Results

### Characteristics of Study Participants

Among 19,096 illness episodes reported by the 3,620 enrolled participants, 16,508 (86.4%) reported by 3,549 participants were linked to a swab that was tested for hMPV and met analysis criteria. Median follow-up time was 1.3 years per participant (IQR follow-up = 0.9–1.5 years; 4,072 person-years total). Among those included, median age at enrollment was 17 years (IQR = 9–41 years), 2,072 (58.4%) participants were female, and 2,496 (70.3%) were non-Hispanic White. Overall, 2,450 (69.0%) participants had an annual household income ≥$100,000, and 3,307 (93.2%) reported living in households with three or more members. The median number of household members included in the analysis was three (range = one to eight), with a median of two adults (range = one to four) and two children (range = one to six) per household.

During the follow-up period, 306 symptomatic hMPV infections were identified among 221 children (aged 6 months–17 years) and 85 adults (aged 18–49 years) (Table 1); 186 (60.8%) detections were of only hMPV, and 120 (39.2%) were detections of hMPV and other pathogens. No participants had repeat infections during the study period. Among 293 (95.8%) symptomatic hMPV infections in households with multiple members included in the analysis, 101 (34%) occurred within 7 days of another symptomatic infection in the same household.

**TABLE 1:** Characteristics associated with incidence of symptomatic human metapneumovirus infection — CASCADIA community cohort (N = 3,549), Oregon and Washington, June 2022–March 2024

Characteristic	Total cases/Total person-years	Incidence* (95% CI)	Hazard ratio (95% CI)	
			Unadjusted	Adjusted^†^
**Overall**	**306/4,072**	**7.5 (6.7–8.4)**	**—**	**—**
**Age group during follow-up**				
6–23 mos	**10/74**	13.5 (7.3–25.1)	3.6 (1.8–7.1)	3.7 (1.8–7.6)
2–4 yrs	**51/261**	19.5 (14.9–25.7)	5.3 (3.6–7.9)	5.8 (3.8–9.0)
5–11 yrs	**114/999**	11.4 (9.5–13.7)	3.0 (2.2–4.1)	3.1 (2.2–4.5)
12–17 yrs	**46/6,517**	7.1 (5.3–9.4)	1.9 (1.3–2.8)	2.0 (1.4–3.1)
18–39 yrs	**29/6,234**	4.7 (3.2–6.7)	1.2 (0.8–2.0)	1.2 (0.8–1.9)
≥40 yrs	**56/1,462**	3.8 (2.9–5.0)	Ref	Ref
**Sex**				
Female	**183/2,369**	7.7 (6.7–8.9)	Ref	Ref
Male	**123/1,703**	7.2 (6.1–8.6)	0.9 (0.7–1.2)	0.8 (0.6–1.0)
**Race and ethnicity**				
White, NH	**206/2,916**	7.1 (6.2–8.1)	Ref	Ref
Other^§^	**100/1,156**	8.7 (7.1–10.5)	1.2 (1.0–1.6)	1.1 (0.8–1.4)
**No. of persons reported in household^¶^**				
1–2	**18/271**	6.6 (4.2–10.5)	Ref	Ref
3	**61/920**	6.6 (5.2–8.5)	1.0 (0.6–1.7)	0.8 (0.5–1.4)
4	**154/1,995**	7.7 (6.6–9.0)	1.2 (0.7–2.0)	0.9 (0.5–1.5)
≥5	**73/886**	8.2 (6.5–10.4)	1.2 (0.7–2.1)	0.9 (0.5–1.5)
No. of children reported in household**				
0	**5/117**	4.3 (1.8–10.3)	Ref	Ref
1	**76/1,040**	7.3 (5.8–9.1)	1.7 (0.7–4.4)	1.1 (0.4–2.9)
2	**161/2,138**	7.5 (6.5–8.8)	1.8 (0.7–4.4)	1.0 (0.4–2.6)
≥3	**64/776**	8.2 (6.5–10.5)	1.9 (0.8–5.0)	1.1 (0.4–2.8)
**Household income (USD)**				
<100,000	**63/905**	7.0 (5.4–8.9)	Ref	Ref
100,000–199,000	**105/1,518**	6.9 (5.7–8.4)	1.0 (0.7–1.4)	1.0 (0.7–1.5)
≥200,000	**120/1,381**	8.7 (7.3–10.4)	1.3 (0.9–1.8)	1.2 (0.8–1.7)
Prefer not to answer	**18/268**	6.7 (4.2–10.7)	0.9 (0.5–1.7)	0.9 (0.5–1.6)
**Health insurance status**				
Employer or individual	**281/3,576**	7.9 (7.0–8.8)	Ref	Ref
Medicaid, Medicare, or other government insurance	**17/296**	5.7 (3.6–9.2)	0.7 (0.4–1.3)	0.6 (0.4–1.2)
Other^††^	**8/167**	4.8 (2.4–9.6)	0.6 (0.3–1.2)	0.5 (0.2–1.1)
**Underlying health condition**				
No	**217/2,453**	8.8 (7.7–10.1)	Ref	Ref
Yes	**89/1,619**	5.5 (4.5–6.8)	0.6 (0.5–0.8)	1.1 (0.8–1.5)
**Year **				
Year 1 (Jun 19, 2022–Jun 18, 2023)	**235/1,757**	13.4 (11.8–15.2)	4.4 (3.4–5.8)	3.5 (2.6–4.6)
Year 2 (Jun 19, 2023–Mar 30, 2024)	**71/2,315**	3.1 (2.4–3.9)	Ref	Ref
**Q**				
Q1 (Jan–Mar)	**208/1,273**	16.3 (14.3–18.7)	5.0 (3.6–7.0)	4.3 (3.0–6.0)
Q2 (Apr–Jun)	**51/742**	6.9 (5.2–9.0)	2.1 (1.4–3.2)	1.2 (0.8–1.9)
Q3 (Jul–Sep)	**8/860**	0.9 (0.5–1.9)	0.3 (0.1–0.6)	0.4 (0.2–0.9)
Q4 (Oct–Dec)	**39/1,197**	3.3 (2.4–4.5)	Ref	Ref
**Study site**				
Kaiser Permanente Northwest	**129/1,960**	6.6 (5.5–7.8)	Ref	Ref
University of Washington	**177/2,112**	8.4 (7.2–9.7)	1.3 (1.0–1.6)	1.2 (0.9–1.6)

**Abbreviations:** NH = non-Hispanic; Q = quarter; Ref = referent group; USD = U.S. dollars.

* Cases per 100 persons per year.

**^†^** Models were adjusted for age group, sex, race and ethnicity, year, Q of the year, household size, household income, and presence of underlying health conditions. Household size was omitted from the model of the number of children in the household because of collinearity.

^§^ Includes NH American Indian or Alaska Native, NH Asian, NH Black or African American, NH Native Hawaiian or Pacific Islander, Hispanic or Latino (Hispanic), NH multiple races, and preferred not to say.

^¶^ Median of three members enrolled per household (range = one to eight), with a median of two members aged ≥18 years enrolled per household (range = one to four).

** Median of two children aged <18 years (range = one to six) enrolled per household.

^††^ Includes no insurance, other insurance, don’t know, and preferred not to say.

Overall incidence of symptomatic hMPV infection was 7.5 per 100 persons per year (95% CI = 6.7–8.4), peaking during January–March (incidence = 16.3; 95% CI = 14.3–18.7) (Figure). Incidence was highest among children aged 2–4 years (19.5; 95% CI = 14.9–25.7) and lowest among adults aged ≥40 years (3.8; 95% CI = 2.9–5.0). During January–March, incidence among children aged 2–4 years was 41.3 (95% CI = 29.2–58.4) (Supplementary Figure, https://stacks.cdc.gov/view/cdc/177080#tabs-3).

**FIGURE:**
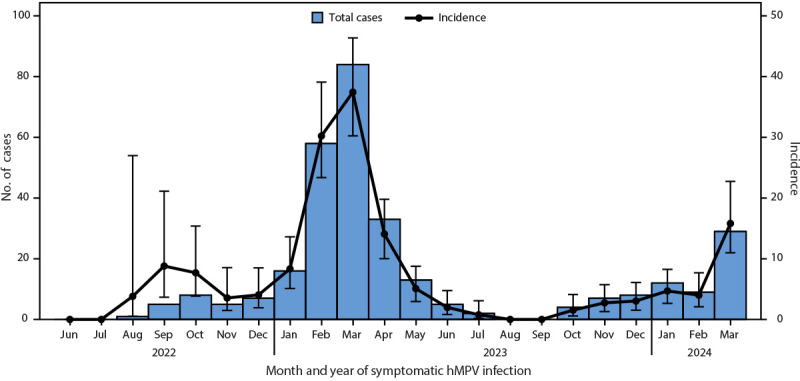
Monthly number of symptomatic human metapneumovirus* cases and incidence^†^ — CASCADIA community cohort, Oregon and Washington, June 2022–March 2024 **Abbreviation:** hMPV = human metapneumovirus. * A participant was considered to have symptomatic hMPV infection if reporting new or worsening illness 0–3 days after collection of an hMPV-positive nasal swab, provided the swab was also collected during the period 2 days before to 7 days after reported illness onset. The date denotes the date of symptomatic hMPV infection. ^†^ Cases per 100 persons per year, with 95% CIs indicated by bars.

In the adjusted survival model, higher incidence was associated with ages 2–4 years (adjusted hazard ratio [aHR] = 5.8; 95% CI = 3.8–9.0), 6–23 months (aHR = 3.7; 95% CI = 1.8–7.6), 5–11 years (aHR = 3.1; 95% CI = 2.2–4.5), and 12–17 years (aHR = 2.0; 95% CI = 1.4–3.1), compared with age ≥40 years (Table 1). Incidence was also associated with season and, compared with October–December, was highest during January–March (aHR = 4.3; 95% CI = 3.0–6.0) and lowest during July–September (aHR = 0.4; 95% CI = 0.2–0.9). The random effects term (θ) that was used to model variation among households was greater than zero (p<0.001), indicating that incidence was correlated within households, and differed among households.***

Symptoms reported among persons with symptomatic hMPV infection included cough (80.4%), nasal congestion (71.9%), sore throat (38.6%), and shortness of breath (7.2%) (Table 2). Compared with adults aged ≥18 years, cough and fever were more likely to be reported among children (odds ratio [OR] = 2.9 and 2.6, respectively), whereas nasal congestion, sore throat, fatigue, and muscle or body aches were less likely to be reported (OR = 0.5, 0.5, 0.4, and 0.4, respectively). Among 252 (82.4%) participants for whom a 14-day post-illness survey was available, 68 (27.0%) missed work, school, or child-care facility attendance, 17 (6.8%) sought medical attention, and two (0.8%) sought in-person health care at a hospital or emergency department. Hazard ratios and illness characteristics were similar when analyses included both hMPV and other pathogens, and when analyses were limited to only hMPV (Supplementary Table 1, https://stacks.cdc.gov/view/cdc/177080#tabs-3) (Supplementary Table 2, https://stacks.cdc.gov/view/cdc/177080#tabs-3).

**TABLE 2:** Characteristics associated with symptomatic human metapneumovirus infection among children aged 6 months–17 years and adults aged 18**–**49 years — CASCADIA community cohort, Oregon and Washington, June 2022–March 2024

Variable	No. (%)			
	Total (N = 306)	Children (n = 221)	Adults (n = 85) (Ref)	Odds ratio* (95% CI)
**Sign or symptom**				
Cough	**246 (80.4)**	189 (85.5)	57 (67.1)	2.9 (1.6–5.2)
Congestion or runny nose	**220 (71.9)**	151 (68.3)	69 (81.2)	0.5 (0.3–0.9)
Sore throat	**118 (38.6)**	76 (34.4)	42 (49.4)	0.5 (0.3–0.9)
Fatigue	**96 (31.4)**	56 (25.3)	40 (47.1)	0.4 (0.2–0.6)
Fever	**72 (23.5)**	61 (27.6)	11 (12.9)	2.6 (1.3–5.2)
Headache	**70 (22.9)**	44 (19.9)	26 (30.6)	0.6 (0.3–1.0)
Muscle or body aches	**45 (14.7)**	24 (10.9)	21 (24.7)	0.4 (0.2–0.7)
Shortness of breath	**22 (7.2)**	17 (7.7)	5 (5.9)	1.3 (0.5–3.7)
Nausea or vomiting	**21 (6.9)**	16 (7.2)	5 (5.9)	1.2 (0.4–3.5)
Diarrhea	**16 (5.2)**	10 (4.5)	6 (7.1)	0.6 (0.2–1.8)
Loss of taste or smell	**11 (3.6)**	6 (2.7)	5 (5.9)	0.5 (0.2–1.5)
Persistent pain or pressure in the chest	**7 (2.3)**	3 (1.4)	4 (4.7)	0.3 (0.1–1.3)
Pale, gray, or blue-colored skin, lips, or nail beds	**1 (0.3)**	1 (0.5)	0 (—)	—
**Care-seeking^†^**	**252**	183	69	—
No care-seeking	**224 (88.9)**	162 (88.5)	62 (89.9)	0.9 (0.4–2.2)
Remote consult	**8 (3.2)**	6 (3.3)	2 (2.9)	1.1 (0.2–5.8)
Medically attended doctor’s office or urgent care	**17 (6.8)**	13 (7.1)	4 (5.8)	1.2 (0.4–4.0)
Pharmacy	**2 (0.8)**	0 (—)	2 (2.9)	—
Visited hospital or emergency department in person	**2 (0.8)**	1 (0.5)	1 (0.5)	0.4 (0–6.1)
Other or unspecified	**3 (1.2)**	3 (1.6)	0 (—)	—
**Absence from work, school, or child care facility**	**252**	183	69	—
Yes	**68 (27.0)**	54 (29.5)	14 (20.3)	1.6 (0.8–3.2)
Days missed from work, school, or child care facility	**76 (31)**	57 (23)	19 (8)	—
Median (range)	**2 (1–8)**	2 (1–8)	1.5 (1–5)	—
**Codetection^§^**				
No	**186 (60.8)**	127 (57.5)	59 (69.4)	Ref
Yes	**120 (39.2)**	94 (42.5)	26 (30.6)	1.7 (1.0–2.9)

**Abbreviations:** Ref = referent group; RSV = respiratory syncytial virus.

* Unadjusted odds ratios for ages 6 months–17 years versus 18–49 years.

^†^ Participant could respond “yes” to one or more categories. Columns might not sum to 100%.

^§^ Codetection of human metapneumovirus with one or more other pathogens: *Streptococcus pneumoniae* (70); rhinovirus (30); adenovirus 1 (13), adenovirus 2 (11); human parainfluenza virus (four); human enterovirus (five); human coronavirus type HKU1 (three), type 229E (one), type NL63 (eight), and type OC43 (four); SARS-CoV-2 (two); influenza A (two) and influenza C (three); and RSV A (one) and RSV B (one).

## Discussion

During June 2022–March 2024, among participants aged 6 months–49 years enrolled in a community cohort, overall incidence of symptomatic hMPV was 7.5 per 100 persons per year. This estimate is consistent with other population-based studies, although previous analyses were generally limited to particular age groups or to medically attended illness (*4**,**5*). hMPV is a leading cause of severe respiratory illness among young children and older adults (*6**,**7*), and pediatric hospitalization rates are comparable to those reported for influenza (*7*). This analysis indicates a high incidence of symptomatic infection in the community that commonly led to missed school, work, or child care facility attendance.

Overall incidence of hMPV varied by age group and season. Incidence rates of symptomatic hMPV infection were highest among children aged 2–11 years (up to 41.3 per 100 persons per year among those aged 2–4 years during January–March). These findings align with serologic evidence from a 2013 study that found the highest rates of hMPV infection among children aged ≥2 years (*8*); however, children aged <2 years might have higher hospitalization rates because of their elevated risk for severe disease (*7*). Incidence over time was consistent with data from laboratory-based surveillance indicating temperate-region predominance during the late winter and spring months (*9*). Lower incidence during the first quarter of 2024 compared with 2023 might also be consistent with reports of biennial variation in timing and transmission (*1*,*9*).

Modeled clustering of incidence by household highlights the role of close contacts in hMPV transmission. Although household size was not associated with infection, comparison was generally limited to households with multiple members. Among households with multiple members enrolled, one third of symptomatic hMPV infections were associated with another symptomatic infection in the same household within 7 days. Underlying health conditions have been linked to severe illness (*1*) but were not associated with incidence of symptomatic infection in this study.

The most frequently reported hMPV symptoms were cough and nasal congestion; shortness of breath was also reported, consistent with involvement of the upper and lower respiratory tracts (*1*). Symptoms varied by age, with children experiencing more fever and cough compared with adults. Because this study was not conditioned on medical attendance or particular symptoms, it might provide a clearer description of symptomatic hMPV infection than that reported in other studies (*1*). Although most participants experienced a relatively mild infection, 27% missed work, school, or child care facility attendance during the 14 days after illness onset, highlighting the impact that even mild infection can have on daily activities.

### Limitations

The findings in this report are subject to at least seven limitations. First, follow-up was <24 months; incidence during April–June was only represented in 2023. Second, incidence might be influenced by changes in seasonality after the COVID-19 pandemic. Third, importance of households as a source of transmission might be underestimated because not all eligible household members were enrolled, and because some infections might be asymptomatic. Fourth, hMPV cases might have been missed because of missed swab collections or assay sensitivity. Fifth, hMPV detection does not prove cause of illness, and other pathogens might have contributed; however, detection usually indicates a causal role (*10*). Sixth, because the CASCADIA cohort only included persons aged 6 months–49 years at enrollment, cases of hMPV illness among younger infants or older adults would not have been captured. Finally, sociodemographics and household size of enrolled participants might not be representative of the general U.S. population.

### Implications for Public Health Practice

Symptomatic hMPV infection is frequently associated with cough or nasal congestion, typically occurring during late winter to spring, with high rates of infection among young children. Although hMPV infections are usually mild, illness can have a considerable impact on daily activities, including work, school, and child care facility attendance. Increased testing for hMPV can identify opportunities for infection control measures and optimize public health surveillance. Understanding hMPV disease incidence can help guide the development and future introduction of vaccines, prophylactic and therapeutic antibodies, antivirals, and nonpharmaceutical prevention products (*2*).

## Footnotes

* Real-Time PCR OpenArray assay tested for hMPV; SARS-CoV-2; influenza A, B, and C; respiratory syncytial viruses A and B; adenovirus; *Chlamydophila pneumoniae* or *Mycoplasma pneumoniae*; human enterovirus (type unspecified); human enterovirus D68; human coronavirus 229E, HKU1, NL63, and OC43; human parainfluenza virus; rhinovirus; and *Streptococcus pneumoniae*.

† Included one or more of the following: fever or chills; cough; shortness of breath or difficulty breathing; fatigue; muscle or body aches; headache, new loss of taste or smell; sore throat; congestion or runny nose; nausea or vomiting; diarrhea; persistent pain or pressure in the chest; and pale, gray, or blue-colored skin, lips, or nail beds.

§ Date of a new hMPV infection was based on reported illness onset, when the onset was ≥30 days since any previous hMPV infection.

¶ Quarters were defined as Q1 (January–March), Q2 (April–June), Q3 (July–September), and Q4 (October–December).

** Includes asthma, chronic obstructive pulmonary disease, heart disease, congenital heart disease, heart failure, Down syndrome, hypertension, liver condition, weak or failing kidneys, cancer, arthritis, stroke, deep vein thrombosis or pulmonary embolism, sickle cell disease or thalassemia, weakened immune system, depression or anxiety, or thyroid issues.

†† An adjusted model evaluating the association between incidence of symptomatic hMPV infection and the number of children in a household did not include household size (number of household members of any age), because of collinearity.

§§ Multivariable regression was not performed because of sparseness of data.

¶¶ C.F.R. part 46.114; 21 C.F.R. part 56.114.

*** The random effects term (θ) was 0.63 and was statistically above the null value (zero).

## References

[1] Branche AR, Falsey AR. Human metapneumovirus [Chapter 159]. In: Bennett JE, Dolin R, Blaser MJ, eds. Mandel, Douglas, and Bennett’s Principles and Practice of Infectious Diseases. 9th ed. Philadelphia, PA: Elsevier Saunders; 2020:2104–9.

[2] Guo L, Li L, Liu L, Zhang T, Sun M. Neutralising antibodies against human metapneumovirus. Lancet Microbe 2023;4:e732–44. 10.1016/S2666-5247(23)00134-9 37499668

[3] Babu TM, Feldstein LR, Saydah S, CASCADIA: a prospective community-based study protocol for assessing SARS-CoV-2 vaccine effectiveness in children and adults using a remote nasal swab collection and web-based survey design. BMJ Open 2023;13:e071446. 10.1136/bmjopen-2022-071446 37451722 PMC10350906

[4] Heikkinen T, Osterback R, Peltola V, Jartti T, Vainionpää R. Human metapneumovirus infections in children. Emerg Infect Dis 2008;14:101–6. 10.3201/eid1401.070251 18258088 PMC2600144

[5] Walsh EE, Peterson DR, Falsey AR. Human metapneumovirus infections in adults: another piece of the puzzle. Arch Intern Med 2008;168:2489–96. 10.1001/archinte.168.22.2489 19064834 PMC2783624

[6] Jain S, Self WH, Wunderink RG, ; CDC EPIC Study Team. Community-acquired pneumonia requiring hospitalization among U.S. adults. N Engl J Med 2015;373:415–27. 10.1056/NEJMoa1500245 26172429 PMC4728150

[7] Williams JV, Edwards KM, Weinberg GA, Population-based incidence of human metapneumovirus infection among hospitalized children. J Infect Dis 2010;201:1890–8. 10.1086/652782 20446850 PMC2873123

[8] Dunn SR, Ryder AB, Tollefson SJ, Xu M, Saville BR, Williams JV. Seroepidemiologies of human metapneumovirus and respiratory syncytial virus in young children, determined with a new recombinant fusion protein enzyme-linked immunosorbent assay. Clin Vaccine Immunol 2013;20:1654–6. 10.1128/CVI.00750-12 23945161 PMC3807191

[9] Haynes AK, Fowlkes AL, Schneider E, Mutuc JD, Armstrong GL, Gerber SI. Human metapneumovirus circulation in the United States, 2008 to 2014. Pediatrics 2016;137:e20152927. 10.1542/peds.2015-2927 27244790

[10] Miyakawa R, Zhang H, Brooks WA, Epidemiology of human metapneumovirus among children with severe or very severe pneumonia in high pneumonia burden settings: the Pneumonia Etiology Research for Child Health (PERCH) study experience. Clin Microbiol Infect 2025;31:441–50. 10.1016/j.cmi.2024.10.023 39489292 PMC13076004

